# Mitofusin2 Induces Cell Autophagy of Pancreatic Cancer through Inhibiting the PI3K/Akt/mTOR Signaling Pathway

**DOI:** 10.1155/2018/2798070

**Published:** 2018-06-26

**Authors:** Ran Xue, Qinghua Meng, Di Lu, Xinjuan Liu, Yanbin Wang, Jianyu Hao

**Affiliations:** ^1^Department of Gastroenterology, Beijing Chao-Yang Hospital, Capital Medical University, Beijing 100020, China; ^2^Department of Critical Care Medicine of Liver Disease, Beijing You-An Hospital, Capital Medical University, Beijing 100069, China

## Abstract

**Aim:**

Pancreatic cancer is one of the most quickly fatal cancers around the world. Burgeoning researches have begun to prove that mitochondria play a crucial role in cancer treatment. Mitofusin2 (Mfn2) plays an indispensable role in mitochondrial fusion and adjusting function. However, the role and underlying mechanisms of Mfn2 on cell autophagy of pancreatic cancer is still unclear. Our aim was to explore the effect of Mfn2 on multiple biological functions involving cell autophagy in pancreatic cancer.

**Methods:**

Pancreatic cancer cell line, Aspc-1, was treated with Ad-Mfn2 overexpression. Western blotting, caspase-3 activity measurement, and CCK-8 and reactive oxygen species (ROS) assay were used to examine the effects of Mfn2 on pancreatic cancer autophagy, apoptosis, cell proliferation, oxidative stress, and PI3K/Akt/mTOR signaling. The expression of tissue Mfn2 was detected by immunohistochemical staining. Survival analysis of Mfn2 was evaluated by OncoLnc.

**Results:**

Mfn2 improved the expression of LC3-II and Bax and downregulated the expression of P62 and Bcl-2 in pancreatic cancer cells. Meanwhile, Mfn2 also significantly inhibited the expression of p-PI3K, p-Akt, and p-mTOR proteins in pancreatic cancer cells. In addition, Mfn2 inhibited pancreatic cancer cell proliferation and ROS production. Assessment of Kaplan-Meier curves showed that Mfn2^−^ pancreatic cancer has a worse prognosis than Mfn2^+^ pancreatic cancer has.

**Conclusions:**

Our finding suggests that Mfn2 induces cell autophagy of pancreatic cancer through inhibiting the PI3K/Akt/mTOR signaling pathway. Meanwhile, Mfn2 also influences multiple biological functions of pancreatic cancer cells. Mfn2 may act as a therapeutic target in pancreatic cancer treatment.

## 1. Introduction

Accompanied with nearly 100% of 5-year mortality rate, pancreatic cancer is one of the most quickly fatal cancers around the world [1]. Although in recent year we have some amazing improvements in the development of surgery, radiation therapy, and chemotherapy, pancreatic cancer still has a desperate prognosis, mainly because of its aggressive biological behavior and late breaking out of symptoms for clinical diagnosis [2]. Traits like that bring a mass of difficulties for therapeutic interventions of pancreatic cancer treatment. One of the main problems for clinical treatment of pancreatic cancer is that we still do not fully understand the pathogenesis and development of this disease. Thus, a deep-going exploration of the malignant essence of pancreatic cancer is urgently needed for the development of novel therapies.

Mitochondria play a significant role in the intermediates needed for macromolecule biosynthesis and the production of ATP [3]. Mitochondria also take part in the activation of signaling pathways. Current evidence suggests that biosynthesis, signaling, and mitochondrial bioenergetics are needed for tumorigenesis [4]. Burgeoning researches have begun to prove that mitochondria play a crucial role in cancer treatment [5]. In the meantime, more and more evidence shows that tumor suppressors and key oncogenes modify the mitochondrial dynamics through significant signaling pathways and that function and mitochondrial mass variables in different tumors and individuals [6, 7].

Mitofusin2 (Mfn2) is a mitochondrial outer membrane protein that plays an indispensable role in mitochondrial fusion, adjusting function and mitochondrial morphology [8]. Research reported that ER stress up-adjusted Mfn2, and genetic ablation of Mfn2 increased cell death during ER stress [9]. In skeletal muscle, Mfn2 regulates the optimal biological properties by maintaining mitochondrial quality control and efficient mitochondrial metabolism [10]. With the knockdown of Mfn2 in Hela cells and a human smooth muscle cell line, impaired autophagic degradation, reduced ATP production, inhibited cell glycolysis and mitochondrial oxygen consumption rate, and suppressed cell proliferation were observed [11].

In recent years, Mfn2 has also shed new light on the area of tumor research. Several studies have found the antitumor effect of Mfn2 in different malignancies, including gastric cancers, breast cancer, hepatocellular carcinoma and urinary bladder cancer [12–14]. The latest study showed that in pancreatic cancer, overexpressed Mfn2 makes cells under apoptotic stress with cleaved caspases. But the cell cycle was not significantly changed with the overexpression of Mfn2. Tumor cells' migration and invasion abilities were inhibited [15]. It is indicated that the overexpression of Mfn2 may become an effective treatment strategy in pancreatic cancer. However, the role and underlying mechanisms of Mfn2 on autophagy of pancreatic cancer cells is still unclear.

In this research, we used adenovirus to deliver Mfn2 to pancreatic cancer cells, so that we can assess the effect of Mfn2 on autophagy. Besides, we uncovered the mechanism of Mfn2-induced autophagy of pancreatic cancer cells. At the same time, we further deeply explore some potential biological mechanisms of Mfn2 by a bioinformatics analyzing.

## 2. Material and Methods

### 2.1. Cell Culture and Passaging

The Aspc-1 cell line was a gift from the cell laboratory of the Beijing Chao Yang Hospital, Capital Medical University. Cryopreserved Aspc-1 cells were thawed and then cultured at 37°C and 5% CO_2_, in proper volume of 10% fetal bovine serum (FBS) in Dulbecco's modified Eagle's medium (DMEM) bought from Gibco (USA). Cells grown to logarithmic growth phase were trypsinized and then passaged.

### 2.2. Adenovirus

Adenovirus encoding the Mfn2 open reading frame (Ad-Mfn2) and control adenovirus were constructed by JI KAI Gene Technology Co. Ltd. (Beijing, China). Aspc-1 cells were cultured for 24 h for synchronization and then incubated with adenovirus at a multiplicity of infection (MOI) of 100 pfu per cell at 37°C for 4 h.

### 2.3. Cell Viability Analysis

Cell viability assay was tested by Cell Counting Kit-8 (CCK-8, Beyotime, China) following the instructions. Cell viability was calculated as follows:

(1) $$\begin{array}{c} Cell\;viability=\frac{A\;450\;nm\;mean\;value\text{ of }infected\;cells}{A\;450\;nm\;mean\;value\text{ of }uninfected\;cells}\times 100\%. \end{array}$$

### 2.4. Caspase-3 Activity Measurement

The activity of caspase-3 was tested using the caspase-3 Activity Assay Kit (C1115, Beyotime, China). The absorbance (A405) was measured using an ELISA reader (BioTek, USA).

### 2.5. Reactive Oxygen Species (ROS) Measurement

Changes in intracellular ROS levels were detected by the oxidative conversion of cell-permeable 2′, 7′-dichlorofluorescein diacetate (DCFH-DA) to fluorescent dichlorofluorescein (DCF). Aspc-1 cells were incubated with Ad-Mfn2 or control media. DCF fluorescence was measured using the FACScan flow cytometer (Becton Dickinson).

### 2.6. Western Blotting Analysis

Western blotting was performed, following that described above [16]. The primary antibody and secondary antibody are listed in Supplementary Table S1 online. Protein bands were visualized using SuperSignal West Pico Chemiluminescent Substrate (Thermo Fisher Scientific, Waltham, MA, USA). Average intensity analysis was used to quantify the protein expression. And the average intensities of each standard protein band were quantified using Photoshop CS5 (Adobe Systems Incorporated), and these results were normalized using GAPDH. The results were column-plotted by GraphPad Prism 7 software.

### 2.7. Immunohistochemical Stains

Immunohistochemistry (IHC) staining was tested as described in previous research [17]. Sections were incubated with mouse monoclonal to Mfn2 (ab56889, Abcam, USA) primary antibodies over one night at 4°C. Then, sections were incubated using the horseradish peroxidase conjugate antibody, while the chromogen used was 2% 3,3′-diaminobenzidine (DAB). As for the histological and immunohistochemical assessment, to analyze Mfn2 expression, according to the number of positive cells, the immunohistochemical results were categorized as follows: +++, positive (>70%); ++, positive (50–70%); +, positive (30–50%); ±, weakly positive (10–30%); and −, negative (<10%). The expression of Mfn2 was assessed blindly and independently by two investigators.

### 2.8. PPI Network Construction and Identification

The Cytoscape app can build a composite gene-gene functional interaction network. The edges are annotated with the results derived from publication or public database [18]. Potential Mfn2-regulated genes were obtained through the GeneMANIA Cytoscape app.

### 2.9. Functional Enrichment Analysis of Mfn2-Regulated Genes

FunRich (Functional Enrichment analysis tool) from ExoCarta (http://www.exocarta.org/) was used to perform analysis. FunRich is an independent software instrument used mainly for interaction network analysis and functional enrichment of proteins and genes. The cut-off standard was *p* < 0.01.

Gene Ontology (GO) mainly contains molecular function (MF), biological process (BP), and cellular component (CC) [19]. The Database for Annotation, Visualization and Integrated Discovery (DAVID, http://david.abcc.ncifcrf.gov/) is a functional annotation tool to understand biological meaning [20]. The Kyoto Encyclopedia of Genes and Genomes (KEGG) [21] provides information about how molecules or genes are networked. The GO-BP, GO-CC, and GO-MF terms were screened with a *p* value of <0.05. Significant enriched KEGG pathways were identified with a *p* value of <0.05.

### 2.10. Survival Analysis of Mfn2 in Human Pancreatic Cancer

OncoLnc (http://www.oncolnc.org) is an instrument for interactively discovering survival correlations. OncoLnc has 8647 patient survival data collected from 21 cancer researches by The Cancer Genome Atlas (TCGA). The total survival of pancreatic cancer patients was analyzed by a Kaplan-Meier plot. The pancreatic cancer patients were separated into two groups on the basis of high or low expression for a particular gene.

### 2.11. Statistical Analysis

Statistical analyses were used by SPSS 16.0 (SPSS, Chicago, IL, USA). *p* values < 0.05 were considered statistically significant. A one-way ANOVA or two-tailed Student *t*-test was performed for intergroup comparison of variance.

## 3. Results

### 3.1. Mfn2 Suppressed Pancreatic Cancer Cell Proliferation

**Figure 1:**
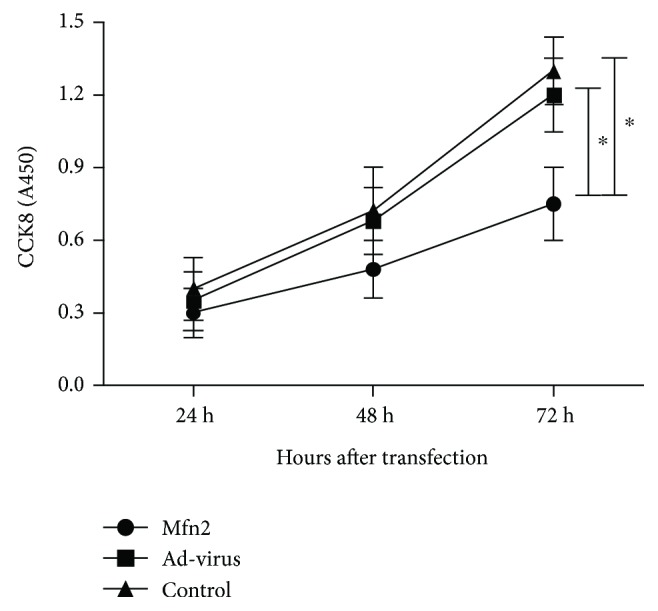
CCK-8 assay was used to verify Aspc-1 cell proliferation (^∗^ *p* < 0.05; *N* = 3).

To further test the effects of Mfn2 on pancreatic cancer cell proliferation, CCK-8 assay was carried out. Compared to the control group, proliferation of Aspc-1 cells was inhibited by Mfn2 overexpression (Figure 1).

### 3.2. Mfn2 Triggers Cell Apoptosis in Pancreatic Cancer

For assessing cell apoptosis in pancreatic cancer cells with Mfn2 overexpression, the expression of Bcl-2 and Bax was measured using Western blotting analysis. Caspase-3 activity was also performed.

**Figure 2:**
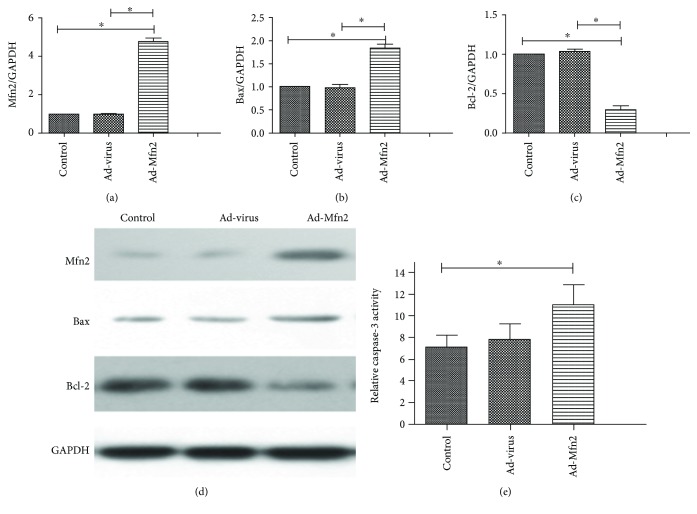
(a) Quantification of Western blots for Mfn2 expressions in the Ad-Mfn2 group compared to control group (^∗^ *p* < 0.05; *N* = 3). (b) Quantification of Western blots for Bax expressions in the Ad-Mfn2 group compared to the control group. (^∗^ *P* < 0.05; *N* = 3). (c) Quantification of Western blots for Bcl-2 expressions in the Ad-Mfn2 group compared to the control group (^∗^ *P* < 0.05; *N* = 3). (d) Western blotting was used to detect the expression of Bcl-2 and Bax to verify cell apoptosis in pancreatic cancer cells. (e) Caspase-3 activity measurement (^∗^ *P* < 0.05; *N* = 3).

The Bax levels were significantly increased in Ad-Mfn2 groups. In addition, Mfn2 significantly reduced Bcl-2 levels of Aspc-1 compared with the control group. Compared with controls, the increased caspase-3 activity was also observed in Ad-Mfn2 groups (Figure 2).

### 3.3. Effect of Mfn2 on Reactive Oxygen Species (ROS) in Pancreatic Cancer

**Figure 3:**
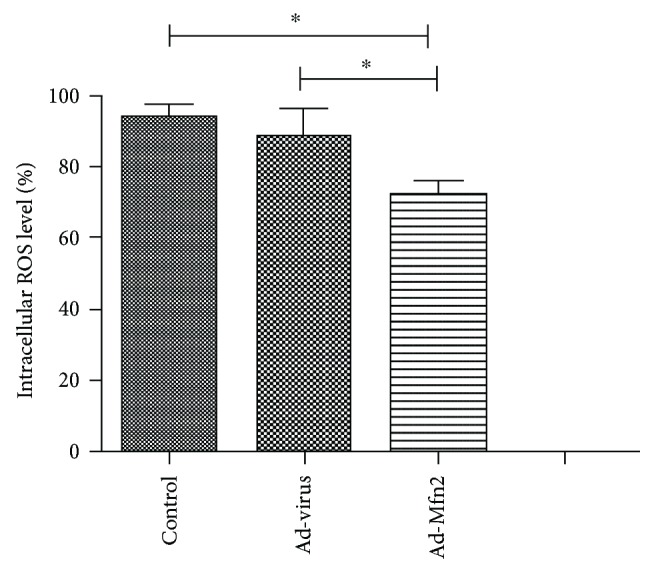
ROS levels were tested using flow cytometry by the DCFH-DA fluorescent probe. There were significant differences between the Mfn2 overexpression group and the control group for ROS level of pancreatic cancer (^∗^ *P* < 0.05; *N* = 3).

ROS levels were performed by flow cytometry in the DCFH-DA fluorescent probe. There was a significant decrease in ROS-positive cells in the Ad-Mfn2 group compared to the control. In the control group, the average rate of DCF-positive cells was 93.12 ± 2.28%, while 71.79 ± 2.42% in the Ad-Mfn2 group contributed to intracellular ROS production (*P* = 0.003) (Figure 3).

### 3.4. Mfn2 Enhances Cell Autophagy in Pancreatic Cancer

**Figure 4:**
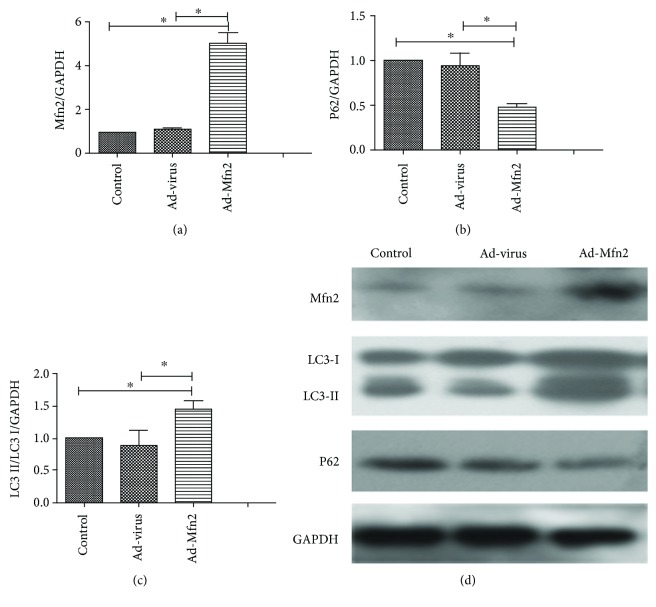
(a) Quantification of Western blots for Mfn2 expressions in the Ad-Mfn2 group compared to the control group (^∗^ *P* < 0.05; *N* = 3). (b) Quantification of Western blots for P62 expressions in the Ad-Mfn2 group compared to the control group (^∗^ *P* < 0.05; *N* = 3). (c) Quantification of Western blots for LC3 II/LC3 I expressions in the Ad-Mfn2 group compared to the control group (^∗^ *P* < 0.05; *N* = 3). (d) Western blotting was used to detect the expression of LC3 and P62 to verify cell autophagy in pancreatic cancer cells.

Western blotting analyses showed that the expression of LC3-II/LC3-I was increased in the Mfn2 overexpression group. There was also a decreased expression for P62 in the Ad-Mfn2 group (Figure 4).

### 3.5. Mfn2 Enhances Cell Autophagy through Inhibiting the PI3K/AKT/mTOR Signaling Pathway

**Figure 5:**
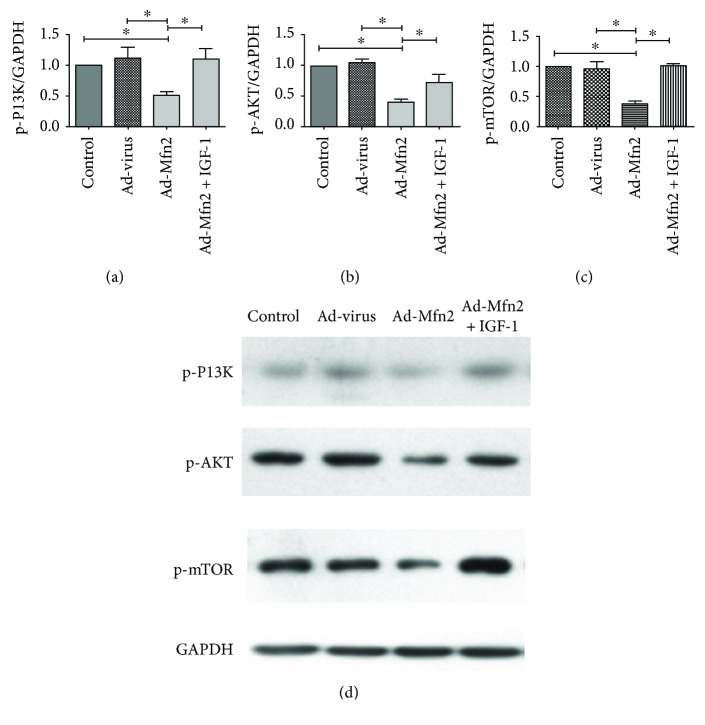
Mfn2 promotes cell autophagy by inhibiting the PI3K/AKT/mTOR signaling pathway. (a) Quantification of Western blots for p-PI3K expressions in the Ad-Mfn2 group compared to the control group and Ad-Mfn2^+^ IGF-1 group (^∗^ *P* < 0.05; *N* = 3). (b) Quantification of Western blots for p-AKT expressions in the Ad-Mfn2 group compared to the control group and Ad-Mfn2^+^ IGF-1 group (^∗^ *P* < 0.05; *N* = 3). (c) Quantification of Western blots for p-mTOR expressions in the Ad-Mfn2 group compared to the control group and Ad-Mfn2^+^ IGF-1 group (^∗^ *P* < 0.05; *N* = 3). (d) Western blotting was used to detect the expression of p-PI3K, p-Akt, and p-mTOR in pancreatic cancer cells.

Next, Western blotting analyses showed that Mfn2 significantly decreased the expression of phosphorylated-PI3K (p-PI3K), phosphorylated-Akt (p-Akt), and phosphorylated-mTOR (p-mTOR) (Figure 5). The expression of p-PI3K, p-Akt, and p-mTOR was significantly decreased in the Mfn2 overexpression group. As an activator of the PI3K/AKT signaling pathway, IGF-1 was given to perform a rescue experiment. After adding IGF-1 in the Ad-Mfn2 group, the expression of p-PI3K, p-Akt, and p-mTOR was significantly increased compared with that of the Mfn2 overexpression group. We consider that Mfn2 induces pancreatic cancer cell autophagy by inhibiting the PI3K/AKT/mTOR signaling pathway.

### 3.6. Association between Mfn2 Expression and Clinic Pathological Factors

**Figure 6:**
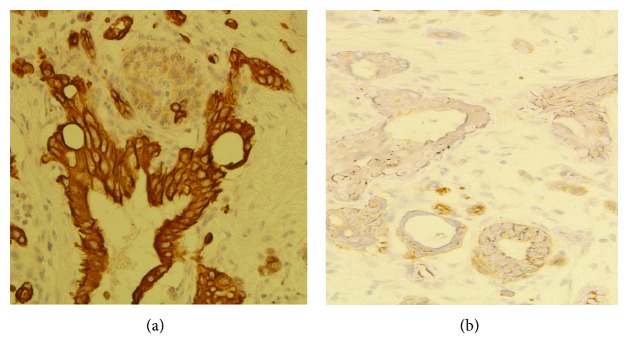
Pancreatic cancer is shown in a pancreas surgical resection specimen cell block with cytoplasmic brown staining with Mfn2 (immunohistochemistry; original magnification, ×400). (b) These cells were almost entirely ASPH^+^ staining. (b) ASPH^−^ staining cells can be found in this case.

The IHC results showed that the Mfn2-positive protein is mainly located in the cytoplasm and is dyed into yellow or yellow granules in the cytoplasm (Figure 6). Mfn2 is expressed both in normal pancreas and in pancreatic cancer tissues. The relationship between Mfn2 expression and clinic pathological factors, including gender, age, differentiation grade, and TNM stage, is shown in Table 1.

### 3.7. Relationship between Mfn2 Immunosubtype and Survival in Pancreatic Cancer

**Figure 7:**
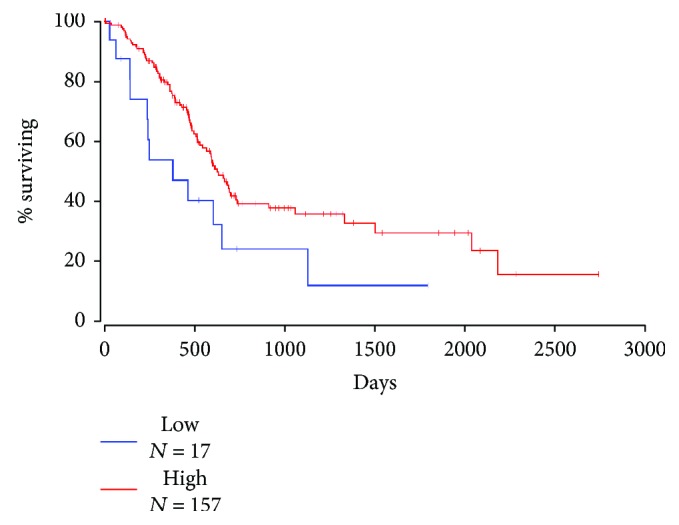
Overall survival curves of patients with pancreatic cancer. The Mfn2 expression curve was calculated according to the Kaplan-Meier method. The red line is the higher expression group (*n* = 157), and the blue line is the lower expression or without group (*n* = 17).

Assessment of Kaplan-Meier curves showed that Mfn2^−^ pancreatic cancer has a worse prognosis than Mfn2^+^ pancreatic cancer has. Patients with an Mfn2-positive expression had a significantly longer survival time than those with an Mfn2-negative expression (*P* = 0.0346, log-rank test) (Figure 7).

Based on the data we observed above, the Mfn2 immunophenotype is closely relevant to the malignant behavior of pancreatic cancer. Mfn2^−^ pancreatic cancer is an aggressive subtype, alongside Mfn2^+^ pancreatic cancer, a less aggressive subtype.

### 3.8. PPI Network Construction

Based on data from GeneMANIA, the PPI network comprised of Mfn2 regulatory genes which were constructed by Cytoscape software (Supplementary Figure S1 Online 1). The network consisted of 20 nodes and 112 links. 112 links included physical interactions (67.64%), coexpression (13.50%), prediction (6.35%), pathway (4.35%), colocalization (6.17%), genetic interactions (1.40%), and shared protein domains (0.59%). The full list of Mfn2 regulatory genes is shown in Supplemental Table S2.

### 3.9. GO Analysis of Mfn2 Regulatory Genes

Following GO analyses for Mfn2 regulatory genes, significant GO terms including cellular component, biological process, and molecular function were collected. Mitochondrion organization and biogenesis was the most significant enrichment of the biological process (*p* < 0.01); mitochondrion was the highest enrichment of the cellular component (*p* < 0.001); and GTPase activity was the highest enrichment of molecular function (*P* = 0.03), as shown in Supplementary Figures S2, S3, and S4 online).

### 3.10. Functional Enrichment Analysis for TFs

The TFs for coexpressed DEGs were significantly enriched in CTCF, OTX1, ELF1, and PITX1 (all *p* < 0.01). CTCF is 11.8% for all transcription factor enrichment analysis. All TFs for coexpressed DEGs are shown in Supplementary Figure S5 online.

### 3.11. KEGG Enrichment Pathways of Mfn2 Regulatory Genes

Following KEGG enrichment analysis for Mfn2 regulatory genes, significant KEGG terms were collected. The pathways enriched were mainly related to viral carcinogenesis by DAIVD (*p* < 0.012) (BAK1, BAX, and UBR4).

## 4. Discussion

Recently, Mfn2 has become a rising star in tumor research [12–14]. In this study, we first proposed that Mfn2 can increase cell autophagy by the PI3K/Akt/mTOR signaling pathway in pancreatic cancer. Mfn2 is considered to perform antiproliferative and proapoptotic functions in pancreatic cancer. Meanwhile, Mfn2 is associated with a good survival rate in pancreatic cancer. Above all, it is indicated that Mfn2 can be a potential clinical therapeutic target in pancreatic cancer.

Autophagy has a complex role in the development of pancreatic cancer, promoting growth of established tumors but suppressing early stages of tumorigenesis. The exact pathways that control the dual roles of autophagy in the pathogenesis of pancreatic cancer, and whether autophagy is efficient or defective, remain to be elucidated [22]. Important mechanisms that could link aberrant autophagy to inflammation in pancreatitis and pancreatic cancer include accumulation of p62 and mitochondrial dysfunction, resulting in increased levels of ROS [23]. In this study, Mfn2 was discovered increasing the cell autophagy by the PI3K/Akt/mTOR signaling pathway in pancreatic cancer. The autophagy declines the ROS production of pancreatic cancer. The effect of autophagy in pancreatic cancer is also the potential mechanism of proapoptotic and antiproliferative functions of Mfn2 in pancreatic cancer.

Mitochondria are highly dynamic organelles, which respond to cellular stress by changes in interconnectedness, overall mass, and subcellular localization [24, 25]. The change in overall mitochondrial mass reflects the balance between the rates of mitophagy and mitochondrial biogenesis [26, 27]. Mfn2 plays an important role in the mitochondrial dynamic fission. In this study, Mfn2 is associated with a good survival rate in pancreatic cancer. This study also indicated that mitochondrial dynamics may be a key aspect of treating cancer.

In this study, we also used bioinformatics methods to analyze potential regulatory genes of Mfn2, aiming to provide valuable information for further biological mechanism elucidation of Mfn2 and provide the groundwork for therapeutic target identification for pancreatic cancer. The pathways enriched were mainly related to viral carcinogenesis by DAIVD, which indicated that Mfn2 is closely linked to the pathogenesis of cancer. This study also has some limitations. Further studies concerning the in vivo effect of Mfn2 for the pancreatic cancer is still required.

## 5. Conclusions

Our finding suggests that Mfn2 induces cell autophagy of pancreatic cancer through inhibiting the PI3K/Akt/mTOR signaling pathway. Meanwhile, Mfn2 also influences multiple biological functions of pancreatic cancer cells. Mfn2 can act as a therapeutic target in pancreatic cancer treatment.

## Figures and Tables

**Table 1:** The relationship between Mfn2 expression and clinic pathological factors.

Number	Age	Sex	TNM	Grade	Stage	Mfn2
1	66	F	T2N0M0	1	I	+++
2	66	F	T2N0M0	1	I	++
3	54	F	T3N0M0	2	II	+
4	54	F	T3N0M0	1	II	+
5	44	M	T3N0M0	2	II	+
6	44	M	T3N0M0	2	II	++
7	59	M	T2N0M0	3	I	+
8	59	M	T2N0M0	−	I	+++
9	63	F	T2N0M0	3	I	+++
10	63	F	T2N0M0	3	I	++
11	53	F	T3N0M0	3	II	+
12	53	F	T3N0M0	3	II	++

## Data Availability

All data used to support the findings of this study are included within the article and supplementary information files.

## Supplementary Materials

Supplementary 1 Supplementary Figure S1: (online 1) PPI network of the Mfn2-regulated gene. The lines represent the protein-protein interaction relationships that correspond to the genes.

Supplementary 2 Supplementary Figure S2: (online) following GO analyses for Mfn2 regulatory genes, significant GO terms for the biological process were collected.

Supplementary 3 Supplementary Figure S3: (online) following GO analyses for Mfn2 regulatory genes, significant GO terms for the cellular component were collected.

Supplementary 4 Supplementary Figure S4: (online) following GO analyses for Mfn2 regulatory genes, significant GO terms for the molecular function were collected.

Supplementary 5 Supplementary Figure S5: (online) functional enrichment analysis of transcription factors (TFs). The transcription factor (TF) enrichment analysis for the Mfn2-regulated gene.

Supplementary 6 Supplementary Table S1: (online) antibodies and conditions used for Western blotting analyses.

Supplementary 7 Supplementary Table S2: Mfn2 regulatory genes obtained by GeneMANIA.

## References

[1] Krempien R., Roeder F. (2017). Intraoperative radiation therapy (IORT) in pancreatic cancer. *Radiation Oncology*.

[2] Birhanu G., Javar H. A., Seyedjafari E., Zandi-Karimi A. (2017). Nanotechnology for delivery of gemcitabine to treat pancreatic cancer. *Biomedicine & Pharmacotherapy*.

[3] Weinberg S. E., Chandel N. S. (2015). Targeting mitochondria metabolism for cancer therapy. *Nature Chemical Biology*.

[4] Guha M., Avadhani N. G. (2013). Mitochondrial retrograde signaling at the crossroads of tumor bioenergetics, genetics and epigenetics. *Mitochondrion*.

[5] Wallace D. C. (2005). A mitochondrial paradigm of metabolic and degenerative diseases, aging, and cancer: a dawn for evolutionary medicine. *Annual Review of Genetics*.

[6] Chen Z.-P., Li M., Zhang L.-J. (2016). Mitochondria-targeted drug delivery system for cancer treatment. *Journal of Drug Targeting*.

[7] Boland M. L., Chourasia A. H., Macleod K. F. (2013). Mitochondrial dysfunction in cancer. *Frontiers in Oncology*.

[8] Schrepfer E., Scorrano L. (2016). Mitofusins, from Mitochondria to Metabolism. *Molecular Cell*.

[9] Ngoh G. A., Papanicolaou K. N., Walsh K. (2012). Loss of mitofusin 2 promotes endoplasmic reticulum stress. *Journal of Biological Chemistry*.

[10] Ainbinder A., Boncompagni S., Protasi F., Dirksen R. T. (2015). Role of Mitofusin-2 in mitochondrial localization and calcium uptake in skeletal muscle. *Cell Calcium*.

[11] Ding Y., Gao H., Zhao L., Wang X., Zheng M. (2015). Mitofusin 2-deficiency suppresses cell proliferation through disturbance of autophagy. *PLoS One*.

[12] Feng X., Zhu K., Liu J. (2016). The evaluative value of Sema3C and MFN2 co-expression detected by immunohistochemistry for prognosis in hepatocellular carcinoma patients after hepatectomy. *OncoTargets and Therapy*.

[13] Ma L., Chang Y., Yu L., He W., Liu Y. (2015). Pro‑apoptotic and anti‑proliferative effects of mitofusin‑2 via PI3K/Akt signaling in breast cancer cells. *Oncology Letters*.

[14] Zhang G. E., Jin H. L., Lin X. K. (2013). Anti-tumor effects of Mfn2 in gastric cancer. *International Journal of Molecular Sciences*.

[15] Sun Q., Wang W. (2016). MFN2 provides antitumor efficacy in pancreatic cancer cells. *Pancreatology*.

[16] Xue R., Yang J., Wu J., Meng Q., Hao J. (2017). Coenzyme Q10 inhibits the activation of pancreatic stellate cells through PI3K/AKT/mTOR signaling pathway. *Oncotarget*.

[17] Xue R., Feng J., Meng Q. (2017). The significance of glypican-3 expression profiling in the tumor cellular origin theoretical system for hepatocellular carcinoma progression. *Journal of Gastroenterology and Hepatology*.

[18] Montojo J., Zuberi K., Rodriguez H., Bader G. D., Morris Q. (2014). GeneMANIA: fast gene network construction and function prediction for Cytoscape. *F1000Research*.

[19] Ashburner M., Ball C. A., Blake J. A. (2000). Gene ontology: tool for the unification of biology. *Nature Genetics*.

[20] Huang d. W., Sherman B. T., Lempicki R. A. (2009). Systematic and integrative analysis of large gene lists using DAVID bioinformatics resources. *Nature Protocols*.

[21] Kanehisa M., Goto S. (2000). KEGG: Kyoto encyclopedia of genes and genomes. *Nucleic Acids Research*.

[22] Gukovsky I., Li N., Todoric J., Gukovskaya A., Karin M. (2013). Inflammation, autophagy, and obesity: common features in the pathogenesis of pancreatitis and pancreatic cancer. *Gastroenterology*.

[23] Perera R. M., Stoykova S., Nicolay B. N. (2015). Transcriptional control of autophagy-lysosome function drives pancreatic cancer metabolism. *Nature*.

[24] Martinou J. C., Youle R. J. (2011). Mitochondria in apoptosis:Bcl-2 family members and mitochondrial dynamics. *Developmental Cell*.

[25] Nunnari J., Suomalainen A. (2012). Mitochondria: in sickness and in health. *Cell*.

[26] Twig G., Hyde B., Shirihai O. S. (2008). Mitochondrial fusion, fission and autophagy as a quality control axis: the bioenergetic view. *Biochimica et Biophysica Acta*.

[27] Youle R. J., Narendra D. P. (2011). Mechanisms of mitophagy. *Nature Reviews Molecular Cell Biology*.

