# Supplementary figures and images for: Mitofusin2 Induces Cell Autophagy of Pancreatic Cancer through Inhibiting the PI3K/Akt/mTOR Signaling Pathway
Source: Oxid Med Cell Longev. 2018 Jun 26;2018:2798070. doi: 10.1155/2018/2798070 (PMC6038474; doi:10.1155/2018/2798070)

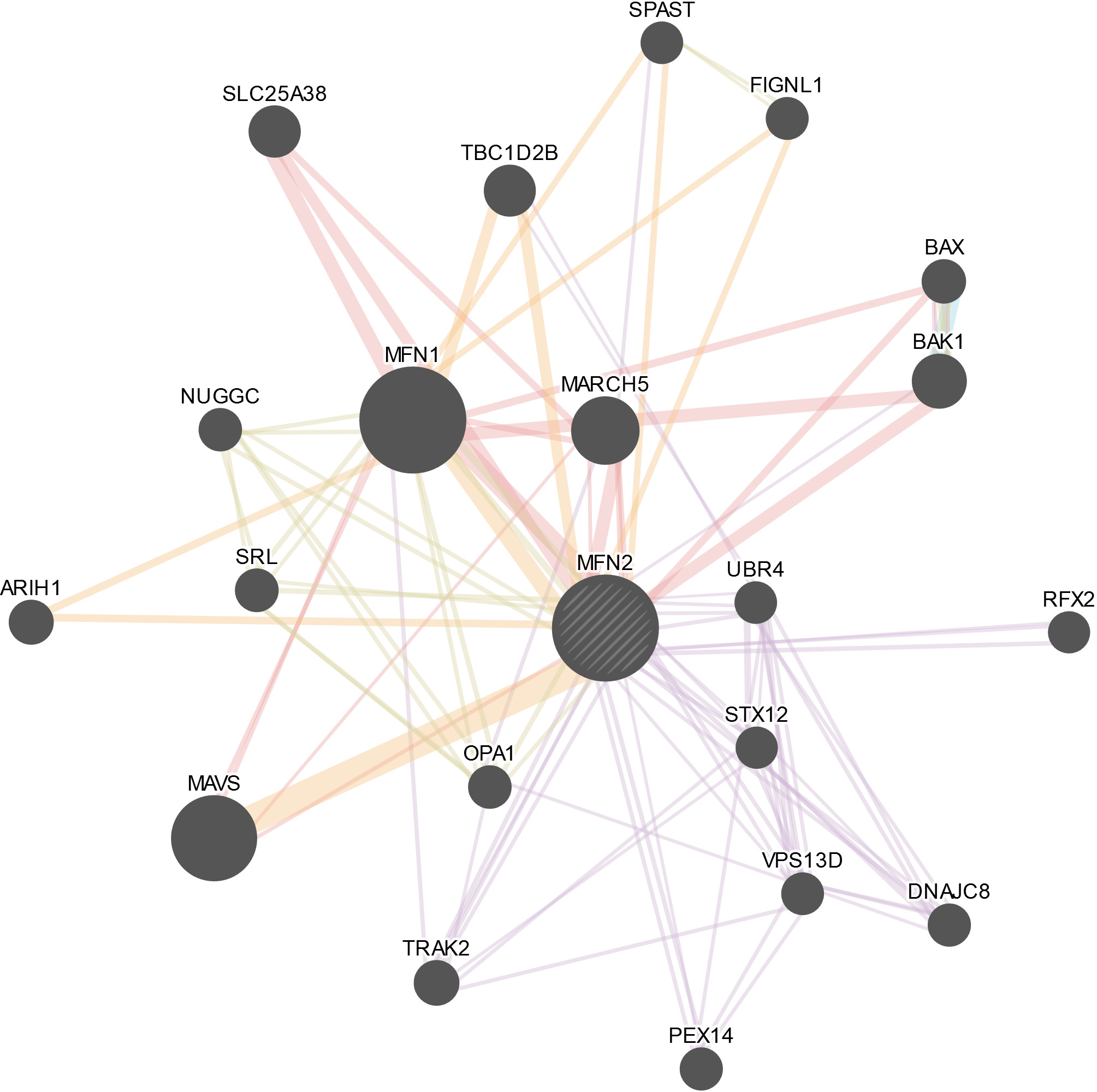

Supplement: Supplementary 1 — Supplementary Figure S1: (online 1) PPI network of the Mfn2-regulated gene. The lines represent the protein-protein interaction relationships that correspond to the genes. [file 2798070.f1.tiff]

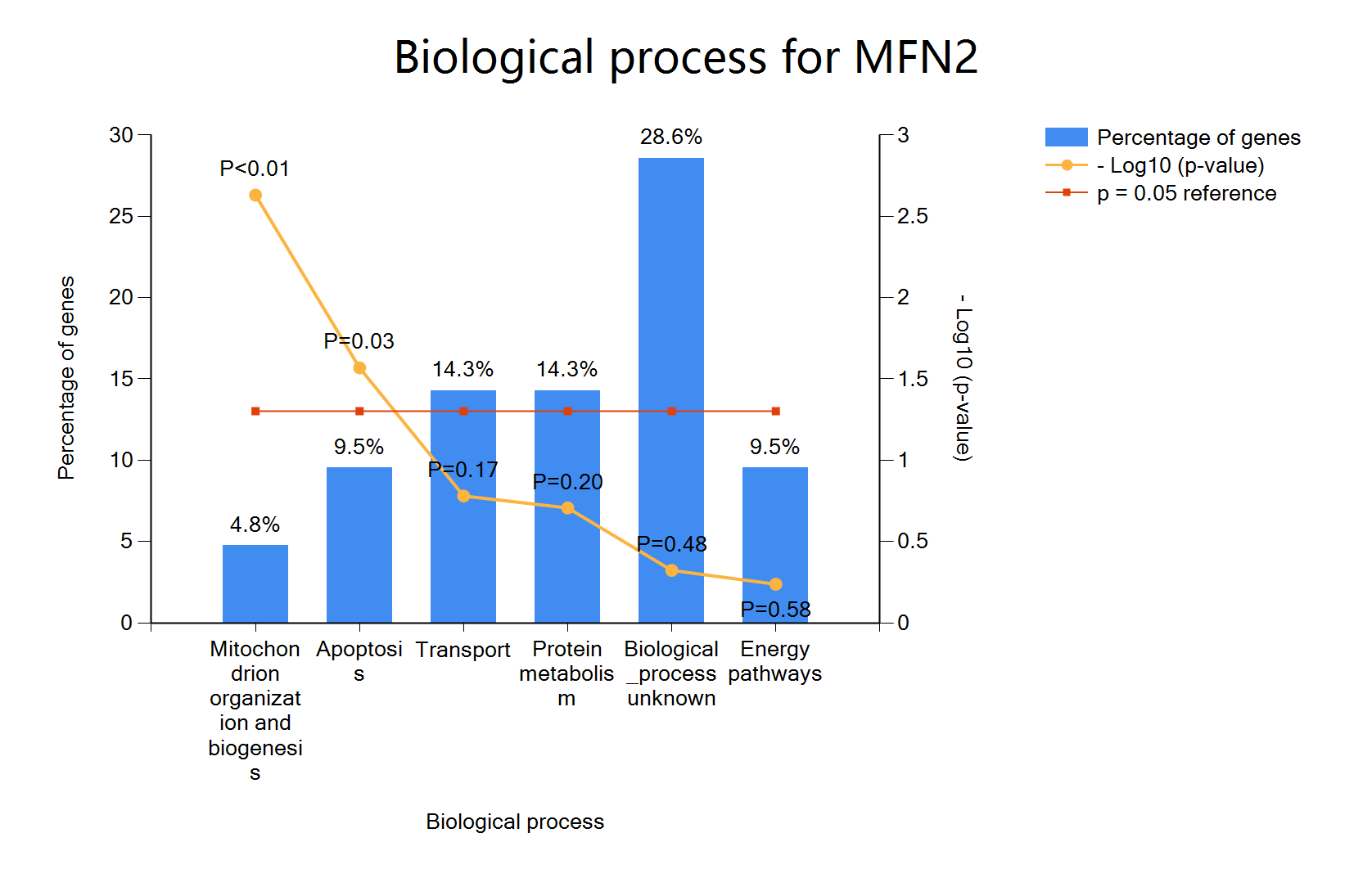

Supplement: Supplementary 2 — Supplementary Figure S2: (online) following GO analyses for Mfn2 regulatory genes, significant GO terms for the biological process were collected. [file 2798070.f2.tiff]

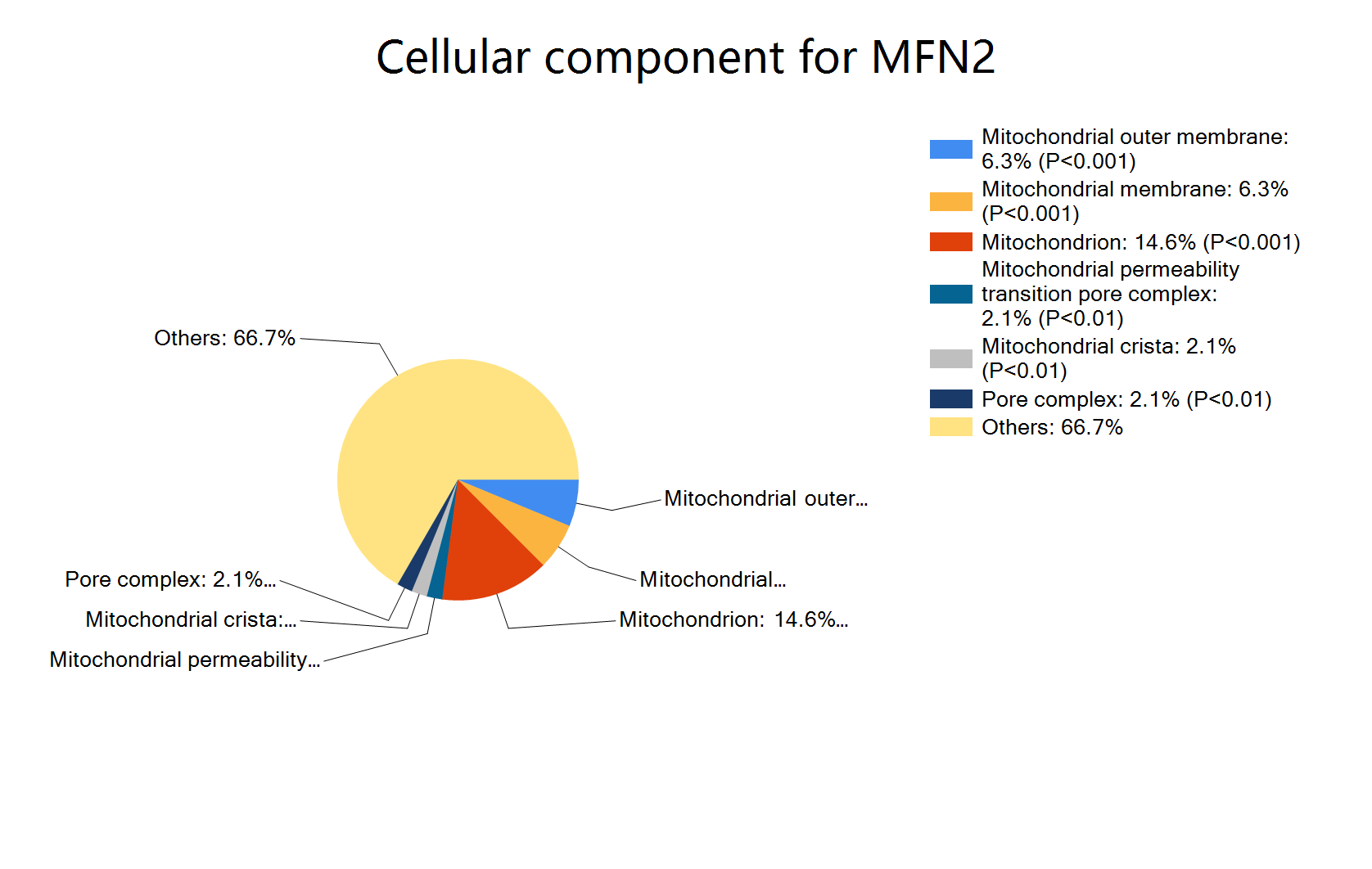

Supplement: Supplementary 3 — Supplementary Figure S3: (online) following GO analyses for Mfn2 regulatory genes, significant GO terms for the cellular component were collected. [file 2798070.f3.tiff]

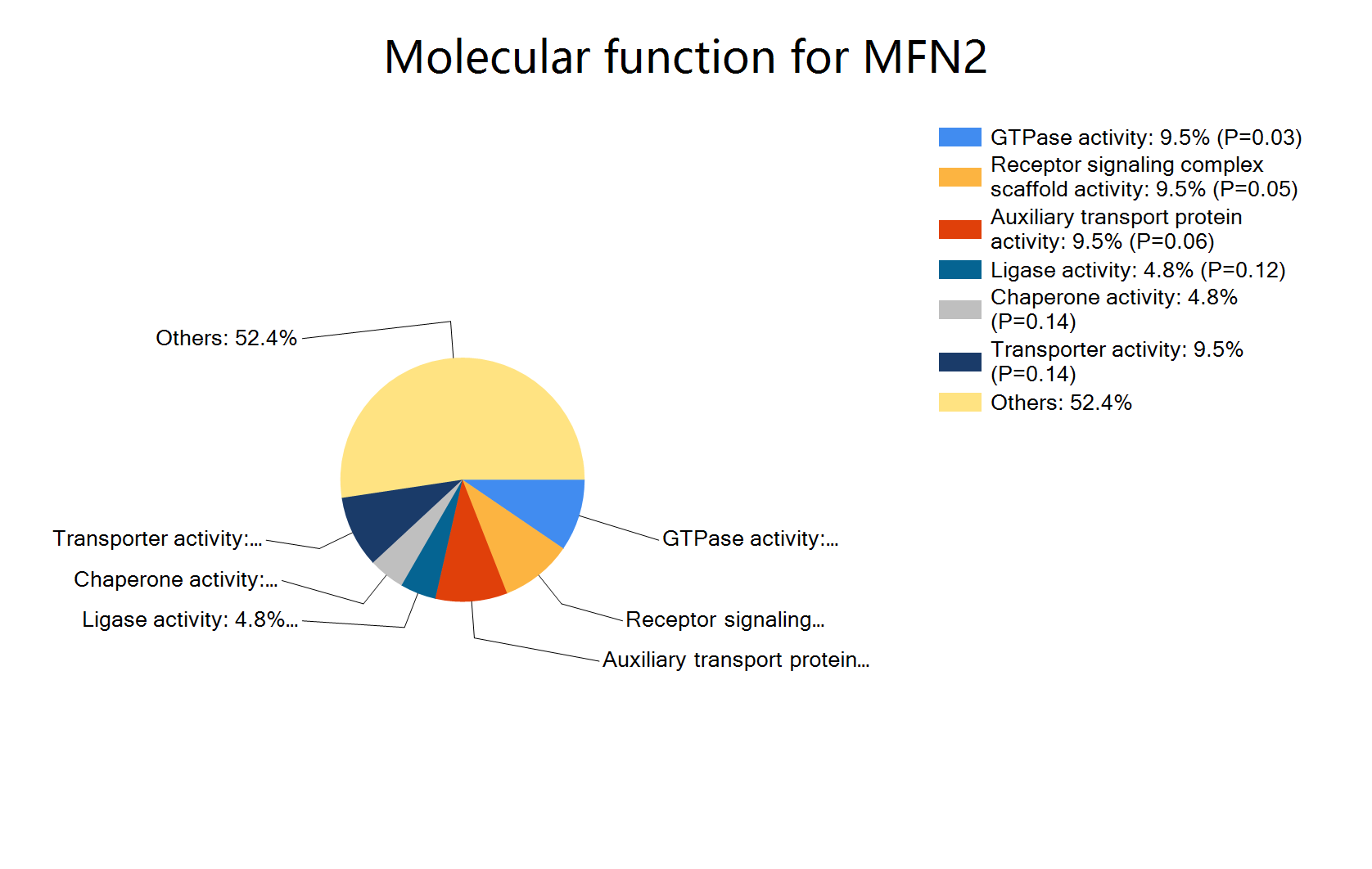

Supplement: Supplementary 4 — Supplementary Figure S4: (online) following GO analyses for Mfn2 regulatory genes, significant GO terms for the molecular function were collected. [file 2798070.f4.tiff]

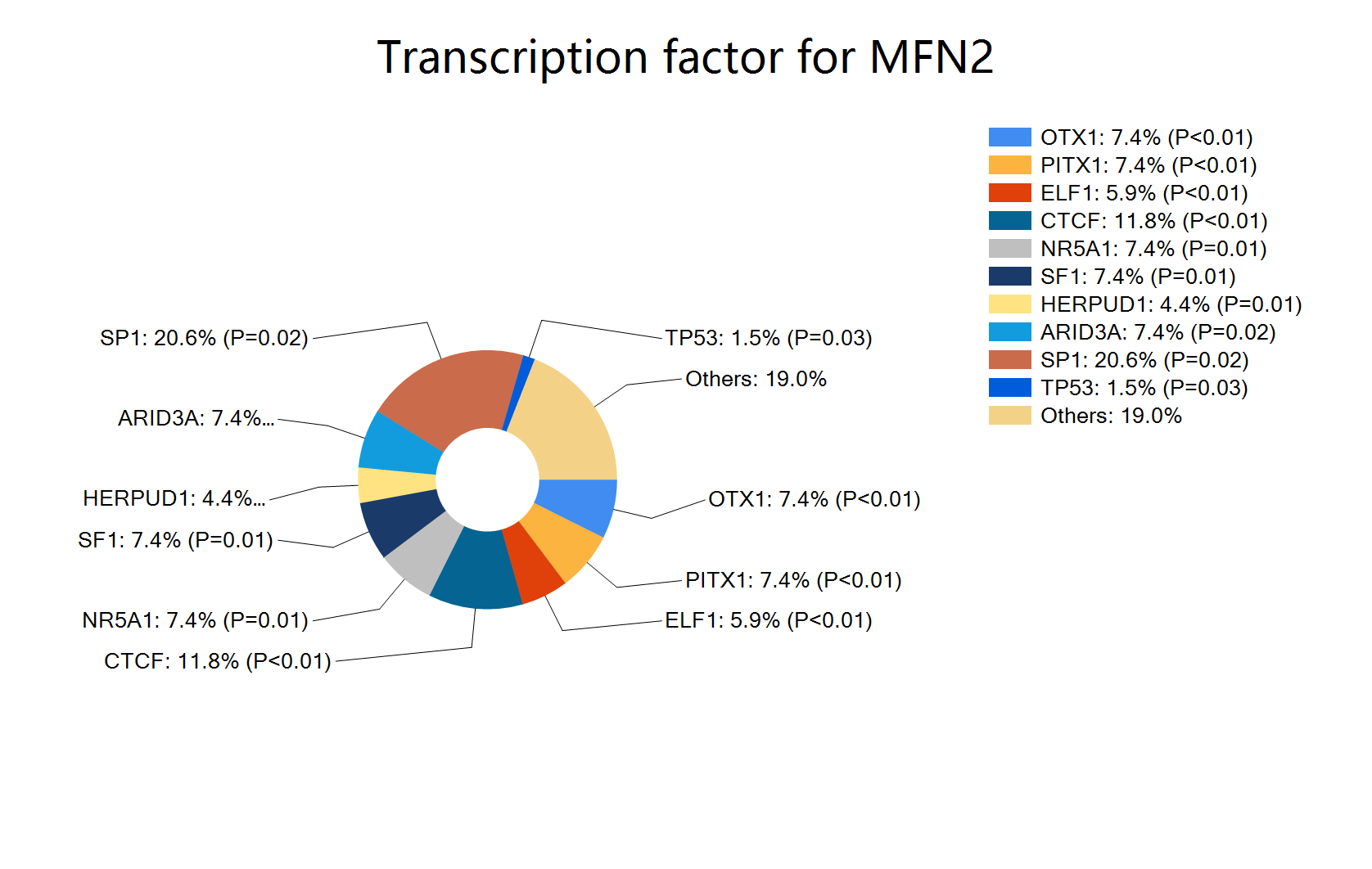

Supplement: Supplementary 5 — Supplementary Figure S5: (online) functional enrichment analysis of transcription factors (TFs). The transcription factor (TF) enrichment analysis for the Mfn2-regulated gene. [file 2798070.f5.tiff]
