# Supplementary material for: Mitofusin2 Induces Cell Autophagy of Pancreatic Cancer through Inhibiting the PI3K/Akt/mTOR Signaling Pathway
Source: Oxid Med Cell Longev. 2018 Jun 26;2018:2798070. doi: 10.1155/2018/2798070 (PMC6038474; doi:10.1155/2018/2798070)
Supplement: Supplementary 6 — Supplementary Table S1: (online) antibodies and conditions used for Western blotting analyses. [file 2798070.f6.docx]

Supplementary Table S1 online

Antibodies and conditions used for western blotting analyses.

| Antibody | Number | Species | Dilution | Source |
| --- | --- | --- | --- | --- |
| Mitofusin 2 | Ab56889 | Mouse monoclonal antibody | 1:1000 | Abcam |
| LC3B | L7543 | Rabbit polyclonal antibody | 1:1000 | Sigma |
| SQSTM1/p62 | #5114 | Rabbit polyclonal antibody | 1:1000 | Cell Signaling Technology |
| GAPDH | #2118 | Rabbit monoclonal antibody | 1:1000 | Cell Signaling Technology |
| Phospho-mTOR (Ser2448) | #2971 | Rabbit polyclonal antibody | 1:1000 | Cell Signaling Technology |
| Phospho-Akt (Ser473) | #4060 | Rabbit monoclonal antibody | 1:1000 | Cell Signaling Technology |
| PI3 Kinase p85 (19H8) | #4257 | Rabbit monoclonal antibody | 1:1000 | Cell Signaling Technology |
| Bax | #2772 | Rabbit polyclonal antibody | 1:1000 | Cell Signaling Technology |
| Bcl-2 (D17C4) | # 3498 | Rabbit monoclonal antibody | 1:1000 | Cell Signaling Technology |
| Cleaved Caspase-3 (Asp175) | #9664 | Rabbit monoclonal antibody | 1:1000 | Cell Signaling Technology |
| Anti-rabbit IgG | #7077 | HRP-linked Antibody | 1:1000 | Cell Signaling Technology |
| Anti-mouse IgG | #7076 | HRP-linked Antibody | 1:1000 | Cell Signaling Technology |
