# Supplementary material for: Mitofusin2 Induces Cell Autophagy of Pancreatic Cancer through Inhibiting the PI3K/Akt/mTOR Signaling Pathway
Source: Oxid Med Cell Longev. 2018 Jun 26;2018:2798070. doi: 10.1155/2018/2798070 (PMC6038474; doi:10.1155/2018/2798070)
Supplement: Supplementary 7 — Supplementary Table S2: Mfn2 regulatory genes obtained by GeneMANIA. [file 2798070.f7.docx]

**Supplementary Table S2**

Mfn2 regulatory genes obtain by GeneMANIA.

| Gene | Description |
| --- | --- |
| MFN1 | mitofusin 1 [Source: HGNC Symbol; Acc:HGNC:18262] |
| MAVS | mitochondrial antiviral signaling protein [Source:HGNC Symbol;Acc:  HGNC:29233] |
| MARCH5 | membrane associated ring-CH-type finger 5 [Source:HGNC Symbol;Acc: HGNC:26025] |
| BAK1 | BCL2 antagonist/killer 1 [Source:HGNC Symbol;Acc:HGNC:949] |
| SLC25A38 | solute carrier family 25 member 38 [Source:HGNC Symbol;Acc:HGNC:26054] |
| TBC1D2B | TBC1 domain family member 2B [Source:HGNC Symbol;Acc:HGNC:29183] |
| TRAK2 | trafficking kinesin protein 2 [Source:HGNC Symbol;Acc:HGNC:13206] |
| ARIH1 | ariadne RBR E3 ubiquitin protein ligase 1 [Source:HGNC Symbol;Acc:HGNC:689] |
| BAX | BCL2 associated X protein [Source:HGNC Symbol;Acc:HGNC:959] |
| SRL | sarcalumenin [Source:HGNC Symbol;Acc:HGNC:11295] |
| OPA1 | OPA1, mitochondrial dynamin like GTPase [Source:HGNC Symbol;Acc: HGNC:8140] |
| NUGGC | nuclear GTPase, germinal center associated [Source:HGNC Symbol;Acc: HGNC:33550] |
| DNAJC8 | DnaJ heat shock protein family (Hsp40) member C8 [Source:HGNC  Symbol;Acc:HGNC:15470] |
| PEX14 | peroxisomal biogenesis factor 14 [Source:HGNC Symbol;Acc:HGNC:8856] |
| FIGNL1 | fidgetin like 1 [Source:HGNC Symbol;Acc:HGNC:13286] |
| VPS13D | vacuolar protein sorting 13 homolog D [Source:HGNC Symbol;Acc:HGNC:23595] |
| SPAST | spastin [Source:HGNC Symbol;Acc:HGNC:11233] |
| UBR4 | ubiquitin protein ligase E3 component n-recognin 4 [Source:HGNC Symbol;Acc:HGNC:30313] |
| STX12 | syntaxin 12 [Source:HGNC Symbol;Acc:HGNC:11430] |
| RFX2 | regulatory factor X2 [Source:HGNC Symbol;Acc:HGNC:9983] |
